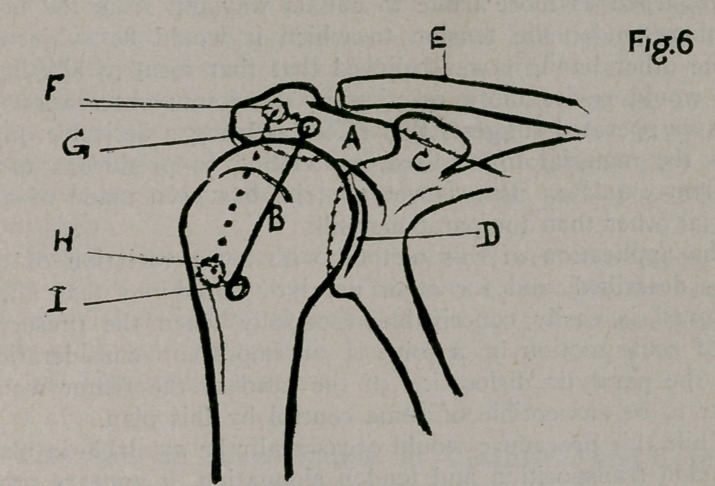# The Use of Intra-Articular Silk Ligaments for Fixation of Loose Joints in the Residul Paralysis or Anterior Poliomyelitis1Read before the Buffalo Academy of Medicine, October 3, 1911.

**Published:** 1912-01

**Authors:** Bernard Bartow, Wm. Ward Plummer

**Affiliations:** Buffalo, N. Y.; Buffalo, N. Y.


### The Use of Intra-Articular Silk Ligaments for Fixation of Loose Joints in the Residul Paralysis or Anterior Poliomyelitis[1]

By BERNARD BARTOW, M.D.
and WM. WARD PLUMMER, M.D.
Buffalo, N. Y.

THE purpose at this time is to present a few observations, based on about a dozen cases of the Residual Paralysis of Poliomyelitis, where artificial ligaments of silk were placed in the interior of loose or "flail" joints, with the intent to limit their excessive mobility, modify malposition and secure stability in the joint sufficient for sustaining body weight.

The rationale of the treatment adopted in these cases may briefly be described as:—"A plan to restrict movement in a joint by means which shall be intra-articular, and still preserve motion in the joint."

This is accomplished by inducing an abundant exudate by means of a reactive inflammation in the joint, excited, first, by the operative trauma, and second, by the presence within the joint of a foreign substance—viz., the silk ligaments to be described. The exudates becoming organized exert a limiting effect on movement, and the strands of silk are of sufficient strength to act as check bands, when so placed, and assist in holding the leg or foot in the weight bearing axis.

The artificial ligaments are both intra-osseous and intra-articular in location, and are passed through the joint by means of tunnels, drilled through the contiguous bone-ends in certain directions. Drawn tightly and tied, while the leg or foot is in the desired position, the silk ligaments hold the articular surfaces in closer coaptation, and at the same time exert a moderate amount of correcting force, for diminishing the malposition.

In addition to their mechanical action, the silk ligaments themselves become the centres of organizing processes, eventually converting them into living tissues, that in time further reinforce the stability of the joint for carrying weight. Originally this plan was adopted to meet the demands of a number of cases presenting extensive paralyses of the lower limbs, all of which required multiple operations, and cobinations of different operative procedures, to secure an effective degree of stability in the joint for the purpose of locomotion.

This plan it was believed would displace the operation of arthrodesis, especially when complete "flail-joint" conditions existed. This was deemed a great advantage, not only because

1. Read before the Buffalo Academy of Medicine, October 3, 1911.

of the frequent failure of arthrodesis, to furnish an efficient form of fixation in the knee or ankle, but also because arthrodesis is an operation inapplicable in many cases, in the lower age scale of childhood.

An extensive experience based on the results of arthrodesis, and the difficulty of obtaining a useful quality of anchylosis from its use, confirmed the views held by the writers, that rigid fixation would probably not follow the introduction of silk ligaments into a large joint. It is important to keep in mind this effect of the plan under consideration, for this form of paralysis, where a measure of regeneration of muscular power is to be looked for in many instances.

For those paralytic conditions requiring combinations of operative procedures, e. g., arthrodesis with tendon transposition, or extra articular silk strands, etc., the plan under consideration is, by comparison a greatly simplified course; it eliminates complicated and prolonged operations, and holds out quite as much promise as any of those mentioned—all of which present a somewhat large percentage of failures.

The material employed for the artificial ligament used in this connection was the parafined silk tendon, prepared in the manner suggested by Lange for tendon elongation. Number 20 Corticelli twisted silk was the size used for both knee and ankle in the earlier operations. Its tensile strength, after operation, was roughly estimated to be sufficient to sustain a direct strain of 125 pounds. For the fixation of the feet in very young children we have recently used number 14 size with satisfactory effect, and greater ease of introduction. In those cases also a very strong Kelly's Trachelorrhaphy needle has been used to introduce the silk, in place of the hand drill, greatly simplifying this detail in the technic.

The technic of the operation in the knee is as follows:

A semicircular flap is raised over the front of the knee, exposing the capsule of the joint, but not opening it. The lateral lines of the incision reach on to the condyles, the lowest part of the incision crossing the inferior border of the patella. The flap is turned up uncovering the patella and condyles. The skin below the line of incision is retracted to expose the head of the tibia as far as its tubercle, and the bone surfaces each side of that point. A hand drill is then pushed obliquely through the inner condyle, from a point near its tuberosity, obliquely across the joint and through the outer tuberosity of the head of the tibia—traversing the patella en route—emerging on the front of the tibia, on a line with the tubercle. (Fig. 1 A. C.) After the drill enters the patella it is made to emerge on its anterior surface near the centre. Fig.1-2 B.) This is the only place where the capsule is punctured. Both ends of an aluminum wire are passed into the eye in the drill and pulled through this section of tunnel as the drill is withdrawn. The drill is again inserted in the patella near the point where it emerged, and pushed through it into the joint, and through the outer tuberosity of the tibia as previously indicated. The drill is again withdrawn with an attached piece of wire. From its point of emergence below the tibial tuberosity it is passed transversely through the compact bone of the tibia, on a line with the tubercle, to the inner side of the tibia (Fig .1 CD.) Again withdrawn with wire attached, the drill is entered at the last point of emergence, and pushed upward through the inner tuberosity of the tibia into the joint, through the patella (with the same detail previously mentioned when traversing that bone) and then through the outer condyle of the femur, to a point opposite the place of first entrance (Fig. 1 DE); from the last point of emergence the drill traverses the compact tissue of the femur to the point of beginning (Fig. 1 EA). The drill when removed from each section of tunnel has a looped piece of wire attached and drawn into it—to be used for pulling through the silk ligament.

Each of the tunnels comes to the surface on the patella in order that the ligament may obtain leverage on that bone (Fig.

2 B.) Other tunnel courses than those indicated may be considered, e. g., drilling in a vertical line through the anterior part of each condyle and through each tibial tuberosity to the front of the tibia, the return tunnel being a half inch to one side and parallel (Fig. 1 FGHI.) (Fig. 2 D E F G). This admits of double anterior ligaments placed each side of the patella. This manner of giving accessory resistance to flexion has only recently been tried by the writers, but it appears to possess advantages that recommend it not only for accessory purposes, but also as a separate and independent mode of limiting knee movement.

The end of the silk is looped into the wire and drawn through the first portion of the tunnel, and so on, through each successive section back to the point of beginning. Drawn tightly as it is pulled through the knee joint it holds the leg in nearly full extension. Before the ends are tied, a small button of bone is removed from the condyle, making a depression in which to bury the knot to prevent pressure on overlying tissues.

The flap having been sutured and the dressings applied, the limb is secured in a plaster of Paris spica extending from the pelvis to the foot, a large fenestrum being made over the knee for inspection and dressing.

The technic of placing silk ligaments in the ankle joint and tarsus, for holding the foot in position, varies from the foregoing only in the anatomic difference of the parts and the type of malposition in the foot.

The tendo-Achilles when shortened is divided as the preliminary step. For the equino-valgus type of deformity, an incision is made through the skin only, extending downward and inward, from a point above the inner malleolus, to the tubercle of the scaphoid (Fig. 3.) The underlying tissues are pushed aside exposing the tarsal and ankle ligaments and the subcutaneous surface of the tibia.

The drill (or the Kelly's needle previously mentioned) is entered on the surface of the tibia above the inner malleolus, passed downward and forward into the ankle joint, penetrating the trochlea of the astragalus, the neck and head of that bone, and thence through the scaphoid, emerging on its internal surface (Fig. 3 a.d.c.b.) The drill being withdrawn, is again entered at this point of emergence on the scaphoid, passed downward and backward through the lateral portion of that bone for a half or three-fourths of an inch. From the last point of emergence the drill is passed upward, backward and outward through the scaphoid, astragalus and tibia, to emerge on the subcutaneous surface of the tibia, a little to one side of the point of first entrance.

A wire is drawn through each section as the drill is removed by means of which the silk ligament is drawn successively through the several tunnels (in the manner described for its introduction into the knee) and the ends tied under firm tension. The purse-string action of the ligament pulls the foot into over-correction, if the foot has previously been manipulated into a state of free mobility.

Fig 4

The use of a strong curved needle in place of the drill is a later modification of the detail in very young cases. Its course, when pushed through the semi-cartilaginous bones of the ankle and tarsus is essentially the same as that indicated for the drill, except that it is brought to the surface at several points, and re-inserted, while traversing the lines of direction which have been indicated for the drill. The needle being large enough to carry the silk no wires are required.

After closing the incision the foot is dressed, and secured in an over-corrected position in a well padded plaster of Paris splint, reaching above the knee, and widely fenestrated over the front of the ankle and foot, to avoid pressure and give access to the dressing.

The technic is only slightly varied to adopt it to the equino-varus type of deformity—the tunnels being then made in the outer side of the ankle and tarsus, and so arranged that the ligament when drawn tightly will exert a flexing and everting action on the foot. (Fig. 4 ABCD).

For the equinus type of malposition—or the "drop-foot", with no associated lateral deviation, the silk ligaments may be passed through tunnels drilled through the tibial end, passing obliquely through the ankle joint and astragalus to the lateral aspect of the and, except for moderate infiltration and tenderness on movement, head of that bone, on each side. These two oblique tunnels are connected at top and bottom by a transverse tunnel in the end of the tibia and head of the astragalus (Fig. 5 ABCD). Only small incisions (¾ inch) are required in the skin and underlying parts over points where the drill enters, and emerges from, the bones. The silk ligaments cross in the anterior part of the astragalus, and when tightened hold the foot in nearly a rectangular position.

Prompt reaction occurs in both knee and ankle joints from the intrusion of the drill, and the presence of the silk ligaments. Effusion with tension and tenderness are quite marked for a week or more after the operation, but with no elevation of temperature. The reaction subsides during the three or four ensuing weeks, the joints are in condition to bear weight with the aid of crutches about the eighth or tenth week after operation.

Fixation of the knee or foot, by this plan, is not complete at the termination of the operation. There occurs a drop of the leg of about 15 degrees before the holding tension of the knee ligaments can be felt. Slight extension and lateral movements in the foot can be made before there will be resistance from the ankle ligament. There is further increase in the amount of these movements when the splints are taken off, and permission given to walk.

Following the removal of the plaster casts in the ankle cases, the feet are protected by valgus or varus shoes, as the case requires, to diminish strain on the silk ligament when the patients are allowed to stand. Following removal of the knee splints, about the tenth week, the only protection the knee receives is from crutches.

The patient is encouraged to stand with the aid of crutches, and bear weight on the knee joint, and make such movements as he is willing to do in the attempt to walk. The purpose of this is to prolong the reactive process in the joint sufficiently to insure an abundant exudate for later organization and thickening of the capsular tissues.

The case presented in this connection fairly represents the conditions for which the procedures just described have been employed, these having been used for the knee and ankle in this instance. The patient who is eight years old, has residual paralyses involving the extensors and flexors of the right leg, causing a drop knee; he also has paralysis of the abductors and flexors of the left foot. The right foot had been a "flail" foot, for which an arthrodesis was performed two and one-half years ago, producing a rigid ankle. The initial attack of polio-myelitis occurred about six years ago.

When first seen by the writer, two and one-half years ago, he could with difficulty stand by holding on to fixed objects, and could walk only when supported by braces aided by crutches, and then only with great effort. At that time an arthrodesis was done at the ankle to secure a rigid foot on which to stand.

In February of the present year the right knee was operated in the manner herein described, and three weeks later an operation of a similar character was made for the relief of the left foot.

This patient was the first case for which this operation was done.

Four months after operation, the knee from free tenderness on weight bearing in the standing position. It was only when tension was placed on the silk ligaments by holding the leg unsupported in extension that there was complaint of pain.

Movement in the knee then permitted it to drop to about twenty degrees from the straight position. With this amount of motion the patient could maintain the straight position of the leg with the higher muscles, and his manner of placing weight on his knee joint.

Except for having a nearly rigid ankle on that side, locomotion was quite easy of performance with slight assistance. At the end of nine months the angle to which the leg dropped increased to about 80 degrees but with no less of the stability which had been secured. During that period there had been marked thickening of the capsular tissues of the knee and ankle from organization of inflammatory exudates.

The patient was placed on his feet, to walk with the aid of crutches, ten weeks after the first operation. The knee and the left foot have been protected by crutches and the valgus shoe during the intervening time, until two months ago, when the patient showed sufficient control to partially discard crutches.

The X-ray appearance of the knee and ankle shows only cloudiness due to exudates—no traces of the silk ligaments being visible.

The ankle and feet after operation are less sensitive to body weight and movement than the knee. The feet remain well within the weight-axis of the limb; and but for the fact that many of these patients with paralytic equino-valgus develope knock knee it would not be difficult to guard the feet against relapse of malposture.

A further application of this idea was adapted to paralysis of the deltoid, in the case of a child five years old who had poliomyelitis three years prior to the time of operation. The object sought was to remove from the deltoid the dragging weight of the arm, which opposed all efforts at regeneration in that muscle and permit arm movements by means of the scapular muscles.

Accordingly the humerus was suspended to the acromion by silk ligaments placed in the tuberosity of the humerus, passing through the joint and through the acromion and tied on the superior surface of that process. The effect of this was to draw the end of the humerus close to the inferior surface of acromion, and obliterate the gap ordinarily seen and felt below the tip of acromion in such instances.

The operative detail (Fig. vi) in this procedure is simple, consisting of:—1st. An incision on the outer aspect of the shoulder from the top of the acromion to about the line of attachment of the deltoid in the humerus, exposing that muscle. 2nd. The drill is passed directly through the muscle fibres to the bone, at the base of the greater tuberosity. It is then pushed upward through the tuberosity into the joint and thence upward through the acromion internal to the insertion of the deltoid; a wire is drawn through the bones as the drill is removed. 3rd. A second penetration of these bones is made with the drill about ¾ inch external to the first, and parallel to it, and a wire also inserted on withdrawing the drill (Fig. vi. FGHI.) 4th. A No. 14 paraffined silk ligament is then pulled upward through the first drill-tunnel. The upper end of the silk is then threaded into a Kelly's needle and passed under the deltoid, close to the periosteum, to the second drill puncture in the acromion. The same

Fig.6

procedure is also adopted to carry the lower end of the ligament under the deltoid at the base of the tuberosity, after which it is attached to the wire and drawn upward through the bones, both ends being tied on the acromion while the head of the humerus is pushed upward and the arm abducted. After suturing and dressing the incision the arm is secured in abduction by an axillary pad and shoulder bandage. The holding power of the ligaments was at once evident. Reaction in the joint was uneventful and practically painless.

While no very definite conclusions can as yet be drawn from the limited experience in the few cases that have been operated, and the short interval of time that has elapsed, it nevertheless appears probable that this procedure has a place of value among the numerous methods in use for the fixation of paralytic joints. It does not, like arthrodesis, involve mutilation of the joint, and does not, like the latter, cause anchylosis. It appears practicable also to employ it in complete paralysis of the leg and foot groups in children of ages unsuitable for arthrodesis. As it produces only limitation of movement, the possibility of regeneration occurring in some of the impaired muscle groups would not be interfered with as in a joint which had been completely anchylosed. That there is little to apprehend from the introduction into large joints of an aseptic foreign substance like the silk ligament is abundantly attested by the case here shown, and by many others not presented at this time.

The paraffined silk tendon of Lange was given the preference in these operations in view of the use that had been made of it for somewhat analagous conditions. The uncovered silk was regarded as more liable to cut its way out from the bone fastenings under the tension to which it would be subjected. On the other hand, it was believed that that form of silk ligament would excite more reaction in the joint; observation of the cases operated suggests that effect as being a desirable quality in the material to be used, as tending to produce a more abundant exudate. But as yet no trial has been made of any material other than the paraffined silk.

The application of this method with some variation of the details described, and for other paralytic conditions than those mentioned is easily conceivable, especially when the preservation of some motion in a joint is an important consideration. Even the paralytic dislocation of the head of the femur would appear to be susceptible of some control by this plan.

While this procedure would occasionally be available in place of tendon transposition and tendon elongation, it appears probable that it may, to a large extent, become a substitute for arhrodesis. In the milder forms of paralytic deformity of the feet, it suffices for holding them in good functionating positions, without transposition of intact muscles, during a period when regeneration may be expected to occur. Its simplicity as compared with the problems of tendon transposition and tendon elongation, is one of the advantages which would often recommend it for preference.

It also offers an opportunity to repeat the operation in the same joint, if necessary, without detriment to the improvement which may have followed an earlier attempt to limit its movement. If, on the other hand, it were evident that this procedure restrained the joint detrimentally in its movements, or the recovery of function or development, it would be easy to remove the restraint exercised by the silk ligament by dividing it anywhere in its continuity, or even withdrawing it altogether.

No tendency has thus far been shown for the silk ligaments to cut their way out of the joint, or cause sloughing of the tissues in contact with them. They become looser, however, with time and the use of the limb. As they cannot stretch, the chief source of looseness is probably from absorption of the bone in which they are buried at points where tension is greatest.

What the ultimate disposal of these artificial ligaments will be, by the tissues in which they are imbedded, and their effectiveness for the purpose intended may, in part be conjectured from our knowledge of their behavior when placed in other tissues, for different conditions from the present, as, e.g., the elongation of tendons, etc.

The more recent and somewhat radical use we have made of this agent suggests that its presence in the bones and joints under aseptic conditions is benign and harmless; that it may be utilized in the manner described to modify instability in joints designed to carry weight when the weight carrying force has been impaired or lost; and further, that it may have even a wide application for securing immobilization, temporary or permanent, in various bony structures when such a condition is desirable.

Further experience with this method, and observation of the time factor, following the use of parts so treated, are necessary to establish definitely the value of the facts that have here been only briefly and superficially presented.

481 DELAWARE AVENUE.

## Figures and Tables

**Fig 1. f1:**
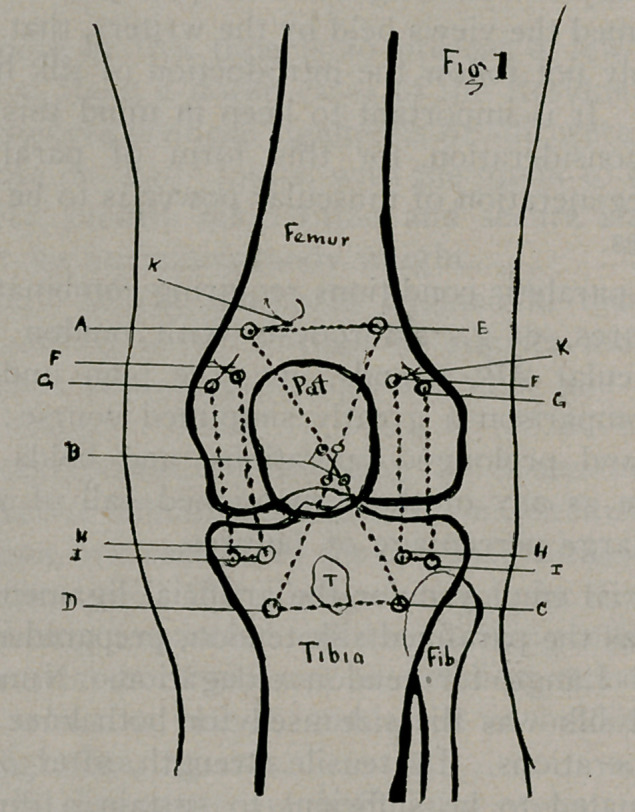


**Fig 2. f2:**
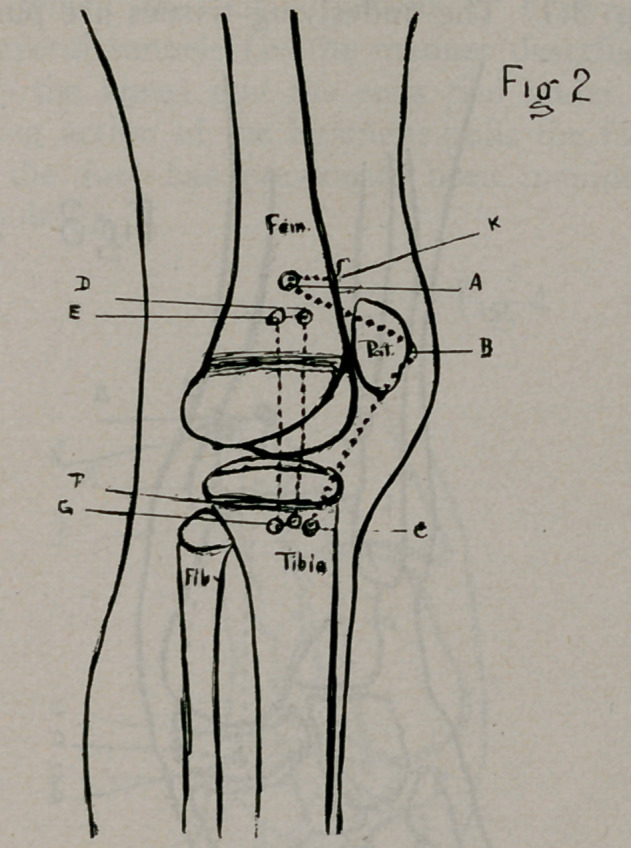


**Fig 3. f3:**
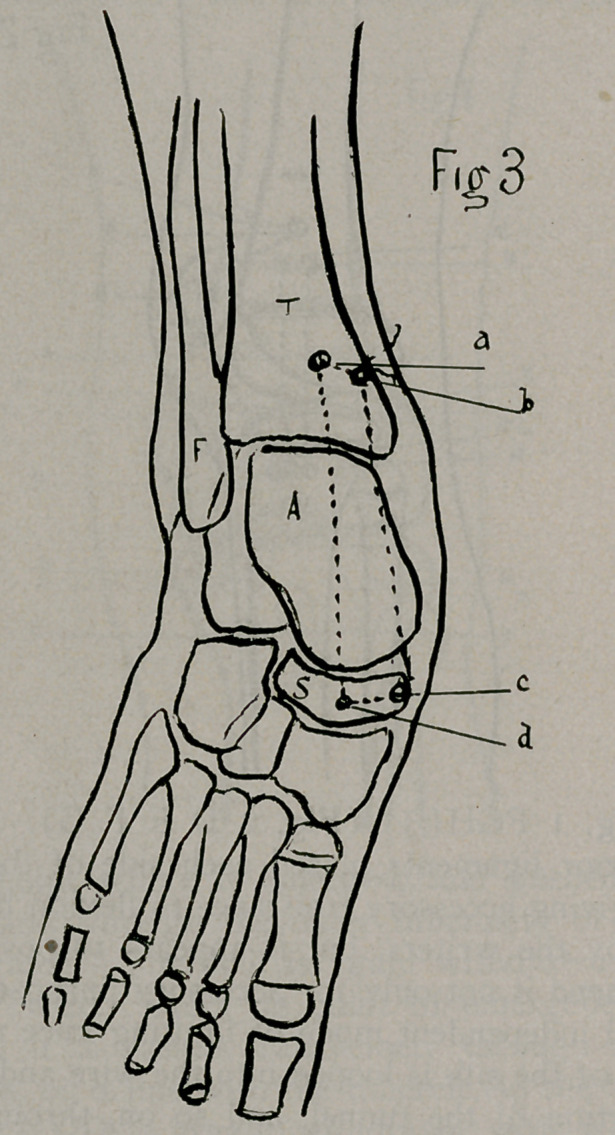


**Fig 4. f4:**
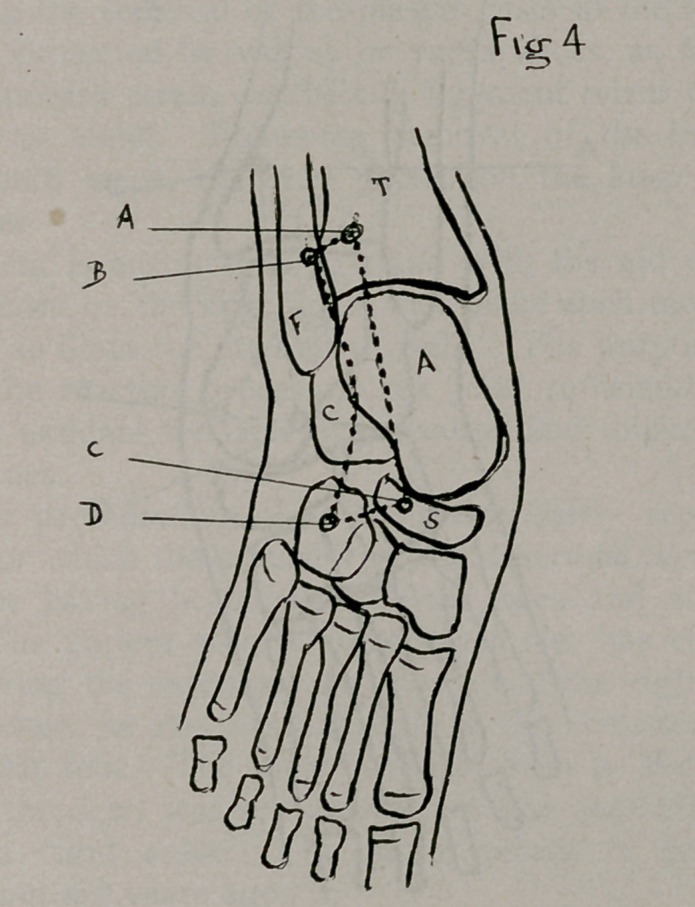


**Fig V. f5:**
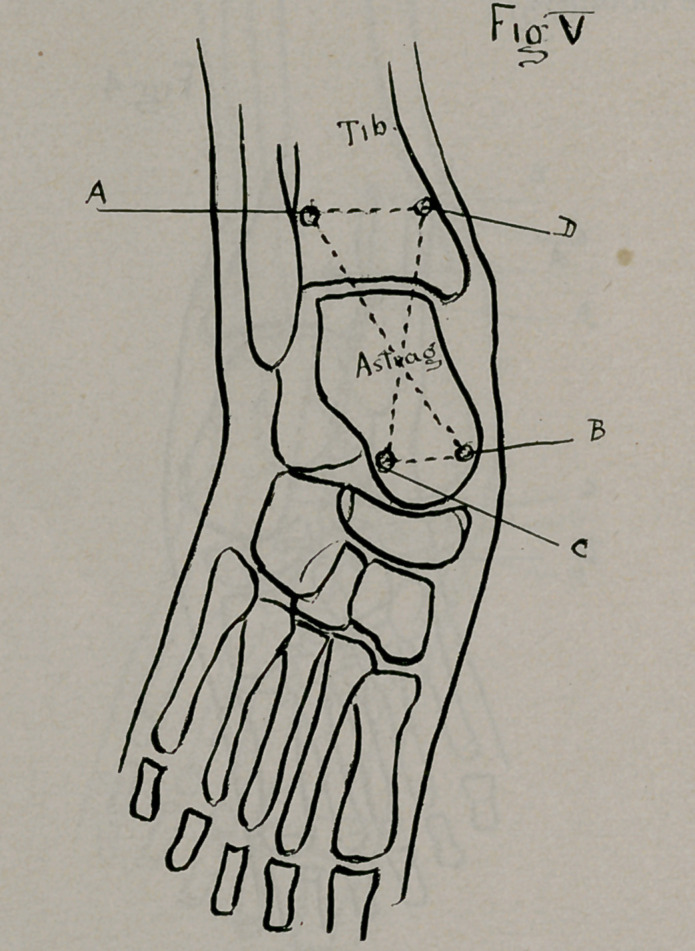


**Fig 6. f6:**